# Supplementary material for: Drug repurposing for aging research using model organisms
Source: Aging Cell. 2017 Jun 16;16(5):1006–15. doi: 10.1111/acel.12626 (PMC5595691; doi:10.1111/acel.12626)
Supplement: Supplementary file 7 — Data S1 Zip‐Archive of all report cards. [file ACEL-16-1006-s007.zip › RC_RX4.pdf]

RX4

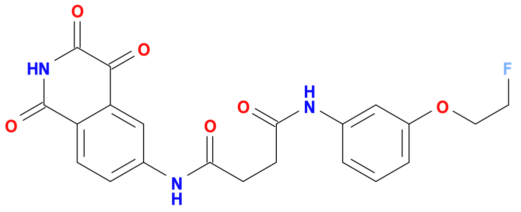

Database identifiers

ChEMBLCompound	CHEMBL1235767

Ranking

	Rank	Score
Drosophila	540/697	0.215
C. elegans	589/591	0.0

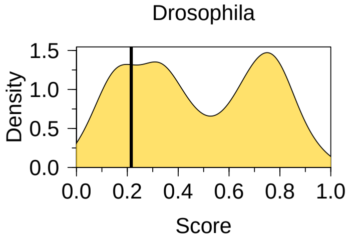

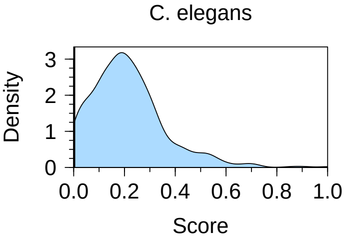

	Ageing implication	Domain conservation	Binding site conservation	Binding affinity	Bioavailability	Lipinski	Promiscuity	Purchasability	Drug approval	**Total**
Drosophila	0.476	0.982	1.0	0.751	(0.9)	-0.1	-0.0	0.0	0.0	0.215
C. elegans	0.476	0.746	0.462	0.751	0.203	-0.1	-0.0	0.0	0.0	0.0

Names

No synonyms found

Roles

ChEBI entry None has no roles

Status

Approved drug (according to ChEMBL)	No
Number of Rule of 5 violations	2
Binding affinity to original target in log units (RF-Score prediction)	6.1
Burns *C. elegans* bioavailability prediction	-8.26

Compound Target Characteristics

Angiotensin-converting enzyme

Best gene implication in ageing for this target family came from gene M0RB66 via mapping the annotation from Ensembl ENSRNOG00000007467 via mapping the annotation from RGD 2493 annotated in RGD 2014-03-11. Annotation GO 7568 (aging) was Inferred from Expression Pattern

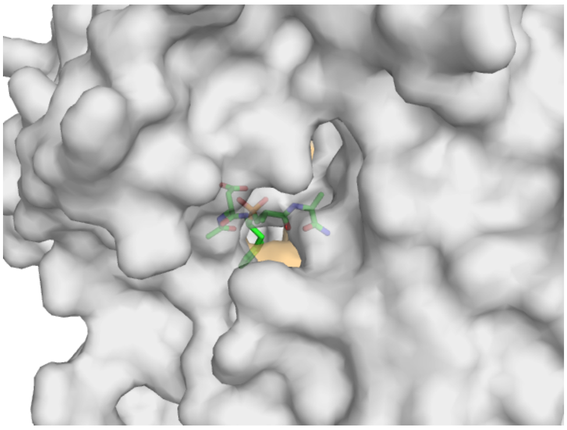

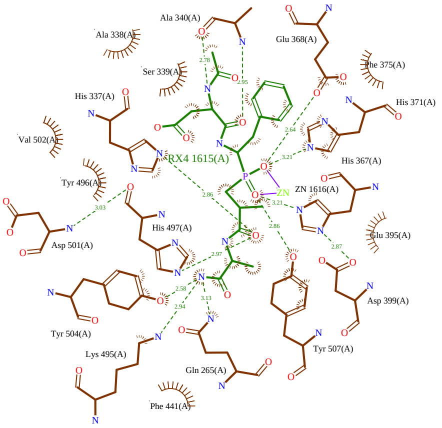

protein	amino acids contacts (binding site)																
PDB:2x97:chainA:Q10714	Q	H	A	S	A	H	E	H	F	E	F	K	Y	H	V	Y	Y
tr:D3DU13:D3DU13_HUMAN	Q	H	A	S	A	H	E	H	F	E	F	K	F	H	V	Y	Y
tr:L7MUH0:L7MUH0_HUMAN	Q	H	A	S	A	H	E	H	F	E	F	K	F	H	V	Y	Y
sp:P12821:ACE_HUMAN	Q	H	A	S	A	H	E	H	F	E	F	K	F	H	V	Y	Y
tr:M0RB66:M0RB66_RAT	Q	H	T	S	S	H	Q	H	Y	E	F	K	F	H	M	Y	Y
tr:Q8K233:Q8K233_MOUSE	Q	H	P	S	A	H	E	H	F	E	F	K	F	H	V	Y	Y
sp:P09470:ACE_MOUSE	Q	H	P	S	A	H	E	H	F	E	F	K	F	H	V	Y	Y
tr:Q3TU20:Q3TU20_MOUSE	Q	H	P	S	A	H	E	H	F	E	F	K	F	H	V	Y	Y
sp:Q10714:ACE_DROME	Q	H	A	S	A	H	E	H	F	E	F	K	Y	H	V	Y	Y
sp:Q18581:ACN1_CAEEL	G	H	A	A	A	S	L	Q	Q	D	K	H	N	S	S	A	T

	whole protein		domain-based		contact-based	
protein	ident	simil	ident	simil	ident	simil
PDB:2x97:chainA:Q10714	1.0	1.0	1.0	1.0	1.0	1.0
tr:D3DU13:D3DU13_HUMAN	0.36	0.63	0.44	0.78	0.94	0.99
tr:L7MUH0:L7MUH0_HUMAN	0.35	0.65	0.45	0.8	0.94	0.99
sp:P12821:ACE_HUMAN	0.2	0.37	0.45	0.8	0.94	0.99
tr:M0RB66:M0RB66_RAT	0.31	0.64	0.4	0.79	0.65	0.85
tr:Q8K233:Q8K233_MOUSE	0.25	0.47	0.44	0.8	0.88	0.97
sp:P09470:ACE_MOUSE	0.19	0.36	0.44	0.8	0.88	0.97
tr:Q3TU20:Q3TU20_MOUSE	0.19	0.36	0.44	0.8	0.88	0.97
sp:Q10714:ACE_DROME	1.0	1.0	1.0	1.0	1.0	1.0
sp:Q18581:ACN1_CAEEL	0.16	0.49	0.23	0.71	0.18	0.46

Ance (FBgn0012037) associated phenotypes

partially lethal - majority live

(Information from FlyBase)

Ance (UniProt:Q10714) annotation

Function: May be involved in the specific maturation or degradation of a number of bioactive peptides. May play a role in the contractions of the heart, gut and testes, and in spermatid differentiation. (PubMed:12591244).

Cofactor: Zn(2+)Note=Binds 1 zinc ion per subunit.;

Enzyme regulation: Inhibited by captopril and, to a lesser extent, by lisinopril, trandolaprilat, fosinoprilat and enalaprilat. (PubMed:7775412, PubMed:8761461).

Biophysicochemical properties: Kinetic parameters: KM=33.5 uM for angiotensin I (PubMed:7775412, PubMed:8761461, PubMed:9839949); KM=53.4 uM for N-acetyl-Ser-Asp-Lys-Pro (PubMed:7775412, PubMed:8761461, PubMed:9839949); KM=2.59 mM for Hip-His-Leu (PubMed:7775412, PubMed:8761461, PubMed:9839949); KM=10.26 mM for Hip-His-Leu-NH(2) (PubMed:7775412, PubMed:8761461, PubMed:9839949); KM=372 uM for (Leu5)enkephalin (PubMed:7775412, PubMed:8761461, PubMed:9839949); KM=1.88 mM for (Leu5)enkephalinamide (PubMed:7775412, PubMed:8761461, PubMed:9839949);

Subcellular location: Secreted, extracellular space.

Tissue specificity: Expressed in vesicular structures in spermatocytes and early spermatids (at protein level). (PubMed:12591244).
Developmental stage: Expressed in the amnioserosa during germ band elongation, shortening and heart morphogenesis. Expressed in midgut throughout embryogenesis.
Ptm: Glycosylated. (PubMed:8761461).
Disruption phenotype: Male flies are sterile. (PubMed:12591244).

(Information from UniProt)

acn-1 (WBGene00000039) associated phenotypes
embryonic lethal, lethal, sterile

(Information from WormBase)

acn-1 (UniProt:Q18581) annotation
Function: Required for larval molting, male tail development, and formation of adult alae. Inactive as a metallopeptidase, due to a lack of active site residues. (PubMed:14559923).
Tissue specificity: Expressed in embryonic and larval hypodermis, in the vulva during organogenesis, and in the ray papillae of the male tail. (PubMed:14559923).

(Information from UniProt)
